# Newer insights to the neurological diseases among biblical characters of old testament

**DOI:** 10.4103/0972-2327.70873

**Published:** 2010

**Authors:** Stephen K. Mathew, Jeyaraj D. Pandian

**Affiliations:** The Leprosy Mission, P.O Motinagar, Masaudha, Faizabad, Uttar Pradesh 224 201, India; 1 Christian Medical College, Ludhiana, Punjab 141 008, India

**Keywords:** Autism, Bible, neurological diseases, stroke, neuropathy

## Abstract

Many people over the years have studied the Bible from a medical point of view offering diagnoses for the symptoms and signs that appear to have afflicted numerous individuals in the Bible. We review the biblical characters in the Old Testament and offer newer insights to their neurological diseases. We first look at the battle between Goliath and David. Interestingly, Goliath probably suffered from acromegaly. We propose autism as a diagnosis for Samson which would precede the first known case of autism by centuries. Isaac was a diabetic, and he probably had autonomic neuropathy. Few verses from the books of I Samuel, Psalms, and Ezekiel reveal symptoms suggestive of stroke. Jacob suffered from sciatica, and the child of the Shunnamite woman in II Kings had a subarachnoid hemorrhage. These instances among others found in the Old Testament of the Bible offer newer insights on the history of current neurological diseases.

## Introduction

The Holy Bible, accepted by Christians all over the world as the word of God, has through the ages remained relevant with its eternal message of love, peace, and hope. Various interpretations of the exact text of the Bible have been arrived by scholars and laypeople alike. The Bible is not just a religious text. It is also a historical account of the Israelites in the Old Testament, to the birth of Christianity in the New Testament. In this paper, we attempt to correlate behavioral patterns of various Biblical figures from the Old Testament with proven neurological diagnoses of today.

## David and Goliath

Over the years, numerous scholars have tried to explain the outcome of this battle with the help of available scientific and medical knowledge. Berginer *et al*. in their publication, have tried to show that Goliath suffered from visual field restriction.[1]

During the battle, as Goliath moved toward David, the young boy moved fast (I Samuel 17:48) while in contrast, Goliath was slow and ponderous.[1–3] It is likely that Goliath suffered from acromegaly.[1,4] His height was “six cubits and a span” (approximately 2.5–3 m).[1] It has been postulated that one of the reasons why Goliath was unable to move fast, apart from his immense physical size, was the arthropathy and myopathy he could have been suffering from, which frequently appear in the later stages of acromegaly.[1,5] Another possible explanation offered by Feinsod was that the giant Goliath suffered a visual defect probably resulting from a pituitary adenoma which allowed David to sneak up unnoticed.[6,7] Further evidence is found in I Samuel 17: 43—“The Philistine said to David, “Am I a dog, that you are coming after me with sticks?””, whereas it is more likely that David carried only one stick; and in I Samuel 17:41—“and the man that bare the shield went before him.” This man who went before the giant was most likely a guide who would indicate the direction from which the enemy was approaching.[1] Berginer *et al*. support the diagnosis of a pituitary macroadenoma causing bitemporal hemianopsia as a result of optic chiasma compression in the suprasellar region.[1] II Samuel 21 offers proof that other members (his brother and some of his sons) from the giant’s family had similar physical appearance.

Further reading of Chapter 17 brings one to verse 49, “And David put his hand in his bag, and took thence a stone, and slang it, and smote the Philistine in his forehead, that the stone sunk into his forehead.” Hirsch *et al*. analyzed this event using contemporary ballistics.[8] Such a high-velocity head injury could have killed even a healthy person from a distance of 30–40 m.[7,8] It can be, therefore, assumed that such a cranial injury was enough to incapacitate the giant Goliath who could have had enlarged frontal sinuses with a thinner frontal bone as a consequence of acromegaly.[9] As a result of the trauma, there could have been an acute occlusion of the Foramen of Monro due to bleeding,[10] which could have led to acute decerebration, and finally the cause of death was decapitation (I Samuel 17:51).

## Samson

The first probable description of autism was made in 1800 by a Frenchman named Jean Marc Gaspard Itard who described deficient communication skills in a young boy named, Victor. It was not until 1911 that the term Autism was coined by Eugen Bleuler, to be later described by Dr. Leo Kanner in 1943. The possibility of Samson having been an autistic would predate the first known case of autism by centuries.

The book of Judges, in Chapter 13 talks about Manoah and his wife, and the child promised to them by God: the child who is named Samson. The subsequent chapters go on to tell us about Samson’s search for a wife, his extraordinary physical powers, which helped him win numerous battles. Various researchers and scholars who have studied the life of Samson have offered Guillain-Barre Syndrome[11] and Myasthenia gravis[12] as possible diagnoses. We would like to offer autism as a possible diagnosis for Samson.

The diagnosis of autism is on the basis of behavioral criteria: qualitative impairments in social and communicative development, with restrictive and repetitive activities and interests.[13] It is written in Judges 13:25 that he had violent movements of the body at times in the camp of Dan between Zorah and Eshtaol. This could have been an instance of recurrent seizure episodes. Two separate studies carried out in New York, and Sweden state that the incidence of epilepsy is greater among children with autism than in the general population.[14,15] However, it must also be stated that the major risk factors for epilepsy among autistics were severe mental deficiency and the combination of severe mental deficiency with a motor deficit,[14] both of which were not evident in Samson.

One of the earliest incidents recorded from Samson’s adult life is the journey to Timnath with his parents where he tears a lion with his bare hands. On his return, he finds a swarm of bees and honey in the carcass of the lion, which he eats, and offers his parents (Judges 14:8–9). Abnormal eating is one of the atypical behaviors noted among children with autism.[16]

Happe states that autistics may exhibit a failure to understand deception (manipulating beliefs) but achieve success on control tasks involving sabotage (manipulating behavior).[13] This may be correlated with the fact that Samson believed his strength lay in his hair, which would be lost if his head were ever shaved (Judges 16:17), and that he succumbed to his wife’s Delilah’s wiles (Judges 16). Throughout Samson’s life, it is seen that he performed extraordinary physical feats. In Chapter 14, he tears a lion with his bare hands “as he would have a rent a kid” (verse 6); in Chapter 15, he kills a 1000 Philistines with the jawbone of an ass; and in Chapter 16 in his final act, he causes the house in which he was held captive to fall by pushing against two pillars that he was tied to, killing more men than he ever did in his life, and in doing so sacrificing his own life. It is possible that Samson was able to perform these feats as he may have been insensitive to pain, which is occasionally seen among autistics.[17] A study of hospitalized individuals carried out in Sweden had reached the conclusion that individuals with autism or autism spectrum disorders are prone to acts of violence.[18]

## Isaac

The first clear allusion to Isaac’s infirmities is seen in genesis 27:1, “and it came to pass, that when Isaac was old, and his eyes were dim, so that he could not see….” Further reading of this chapter brings to light the incident where Isaac is unable to differentiate between his sons Esau and Jacob by the feel of their skin (although Jacob had disguised himself). This leads one to suspect that Isaac suffered from a peripheral sensory neuropathy, as he was unable to feel the difference between human skin and goat skin.

It has been speculated that Isaac could have been a diabetic.[19,20] The arguments in favor of diabetes put forward by Reisenberger[20] and Ginsberg[21] were that both Isaac and his father Abraham looked alike (premature aging of Isaac), Isaac needed a constant source of water (genesis 26 shows that his servants were constantly digging wells wherever they moved to), had no noteworthy physical achievements noted in the bible, was fond of food, suffered visual loss and had impotence (his wife Rebecca delivered Esau and Jacob 20 years after marriage). It is also possible that the autonomic neuropathy resulted from diabetes. All these points urge one to consider that Isaac probably suffered from adult-onset diabetes mellitus, and could be one of the first recorded cases of diabetes.

## Psalm 137 and Ezekiel chapter 3

Psalm 137 was written 500–600 years before the birth of christ during the period of the second temple after the exile.[22] This psalm deals with the punishment for those who forget jerusalem.[23] Resende and cowokers analyzed various language versions of this psalm to offer invaluable insight. The symptoms found in verses 5 and 6 (“right hand shall be paralyzed,” “tongue shall stick to my palate”) offer a diagnosis of stroke with right hemiplegia and motor aphasia.[24,25] A similar passage is found in Ezekiel,[24] where god commands ezekiel to shut himself inside his house (v 24) where god would make “thy tongue cleave to the roof of thy mouth, that thou shalt be dumb, and shalt not be to them a reprover: for they are a rebellious house” (v 26). The following verse goes on to say that god would, later, return the power of speech to the prophet Ezekiel.

## Jacob

Genesis 32:25–32 provides a description of Jacob ’s duel with god (who appeared in the form of an angel). Jacob had camped overnight at a place that later came to be known as peniel, after having sent his family and servants ahead of him, when the angel appeared and wrestled Jacob till daybreak. The angel could not overpower Jacob, and consequently he “touched the hollow of his thigh; and the hollow of Jacob ’s thigh was out of joint” (chapter 32:25). A diagnosis of sciatica appears to be the general consensus,[11,26,27] and the cause for the traumatic monoparesis could have been a posterior dislocation of the hip severe enough to damage the sciatic nerve. If such is the case, the dislocation could have disrupted the blood supply to the femoral head leading to an avascular necrosis of the head of femur.

## The Child of the Shunnamite Woman

II Kings 4 describes an event concerning a Shunammite woman and her son, who was born to her after the prophet Elisha had interceded with God on her behalf:[19] “And he said unto his father, My head, my head. And he said to a lad, Carry him to his mother.[20] And when he had taken him, and brought him to his mother, he sat on her knees till noon, and then died.” Various authors[11,26,28] are of the opinion that this is a case of subarachnoid hemorrhage. Budrys[11] and Tubbs *et al*.[26] propose that the most probable cause for acute subarachnoid hemorrhage is an arteriovenous malformation considering the young age of the child,[26] and the paucity of focal neurological symptoms.[11] It should be noted that the child did not die from the subarachnoid hemorrhage, but gradually lost consciousness and was later resurrected by Elisha (verses 34,35). Loss of consciousness caused by the growing intracerebral pressure is a frequent complication of subarachnoid hemorrhage.[11]

## Nabal

I Samuel Chapter 25 narrates the story of Nabal, the rich cattle breeder, who refused to provide King David some of his sheep (v10). Angered, David then vows to slay Nabal (v13), but Nabal’s wife Abigail intercedes for him (v26) and secures the life of her husband and sons (v34). After a night of heavy drinking, when Nabal heard of this, “his heart died within him, and he became as a stone” (Chapter 25:37), and 10 days later Nabal passes away. A diagnosis of stroke has been offered in this case.[29] Paralysis of the body, followed by death was regarded as two fundamental symptoms of stroke up until the nineteenth century, a diagnosis fortified in this case by the preceding consumption of an excess of alcohol.[30] It is possible that Nabal suffered acute alcoholic cardiomyopathy or cardiac arrhythmia, leading to what could have been a cardioembolic brainstem stroke.

## Conclusion

In conclusion, we offer fresh insights to the neurological diseases among the biblical characters of the Old Testament. Stroke has been described clearly in terms of recognizable symptoms and signs in the books of I Samuel, Psalms, and Ezekiel. The possibility of Samson having been an autistic would predate the first known case of autism by centuries. Suggestions that Isaac could have been a diabetic suffering from resultant diabetic retinopathy and autonomic neuropathy, or that Jacob suffered from sciatica will hopefully spur a deeper interest in the study of Biblical texts from a medical standpoint.
